# Status of cryptosporidiosis in cattle raised by marginalized nomadic communities of Southern Punjab, Pakistan: an observational study

**DOI:** 10.3389/fvets.2026.1820598

**Published:** 2026-05-27

**Authors:** Mian Muhammad Awais, Mohamed Tharwat, Tahir Yaqoob, Masood Akhtar, Abdul Sammad Ali Khan Shirwany, Muhammad Ahmad, Faqir Muhammad, Saleh Alkhedhairi

**Affiliations:** 1 One Health Research Laboratory, Department of Pathobiology, Faculty of Veterinary Sciences, Bahauddin Zakariya University, Multan, Pakistan; 2 Department of Clinical Sciences, College of Veterinary Medicine, Qassim University, Buraidah, Saudi Arabia; 3 University of Baltistan, Skardu, Pakistan; 4 Department of Public Health, Faculty of Medicine and Allied Health Sciences, Times University, Multan, Pakistan; 5 Department of Biosciences, Faculty of Veterinary Sciences, Bahauddin Zakariya University, Multan, Pakistan; 6 Department of Medical Biosciences, College of Veterinary Medicine, Qassim University, Buraidah, Saudi Arabia

**Keywords:** cattle, coproprevalence, cryptosporidiosis, *Cryptosporidium parvum*, ELISA, nomadic communities, risk factors

## Abstract

**Introduction:**

*Cryptosporidium* (*C*.) is one of the most important causes of gastrointestinal disorders in various animal species, leading to significant economic losses in the dairy sector. The parasite has also significant zoonotic relevance, with well-documented cross-transmission between animals and humans. However, data on the status of cryptosporidiosis in scattered nomadic populations is scarce. Therefore, this study was conducted to determine the prevalence and associated risk factors of cryptosporidiosis in cattle raised by socioeconomically deprived nomadic communities in an important agro-ecological zone (Multan) of South Punjab, Pakistan.

**Methods:**

Fecal samples (*n* = 184) were collected from cattle belonging to nomadic communities of study area. Samples were analyzed for the detection of *C. parvum* copro-antigen using commercially available species-specific ELISA kits (IDEXX, USA; sensitivity 98%, specificity 94%). Data on potential risk factors were collected using a pre-designed questionnaire and analyzed using bivariate and multivariable logistic regression analyses.

**Results:**

Data showed that overall prevalence of cryptosporidiosis in target cattle population was 13.59% (*n* = 25/184; 95% CI: 9.03%-19.21%). Bivariate analysis revealed significant association (*p* < 0.05) of cryptosporidiosis with age, health status, history of gastrointestinal problems, physiological status of females, herd size, drinking water source, history of deworming and routine vaccination, and contact with other susceptible animals. Multivariable logistic regression analysis identified age ≤ 1 year (OR = 7.53), contact with susceptible animals (OR = 7.79), small herd size (1–5 heads; OR = 0.21), and history of routine vaccination (OR = 0.17) as significant predictors of cryptosporidiosis.

**Conclusion:**

Findings of this study concluded that *C. parvum* is prevalent in cattle raised by nomadic communities in the study area. Targeted animal health action plans should be developed to control infectious zoonotic diseases, including cryptosporidiosis, by considering nomadic populations as an integral segment of society. The frequent movement of nomads and their animals may facilitate disease transmission across regions. Awareness campaigns are recommended to prevent and control diseases of veterinary public health importance.

## 1 Introduction

Cryptosporidiosis is primarily a gastrointestinal disease which affects both animals and human beings. It is caused by several species of protozoal parasites belonging to genus *Cryptosporidium* (*C*.) and is worldwide in distribution (1). To date, more than 40 species of *Cryptosporidium* have been identified but the most frequently reported species which affect different animal species include *C*. *parvum, C*. *hominis, C*. *canis, C*. *meleagridis, C*. *muris*, and *C*. *felis* (2, 3). It is also one of the emerging human pathogens, particularly among aged individuals, children and patients with severe infections and compromised immunity, where it may lead to life-threatening outcomes (4). Cryptosporidiosis plays a crucial role in reducing productivity of the animals, since this protozoal parasite has a negative impact on the milk yield, feed conversion ratio and growth performance. Although it is a distinct enteric protozoal parasite that infects a wide range of mammalian species, however bovines are the most affected animal species by this parasite. In newborn calves, disease is highly prevalent and results in economic losses due to higher treatment cost and sometimes mortality (5). *Cryptosporidium* infection can lead to severe gastrointestinal tract (GIT) disorders with clinical manifestations including anorexia, nausea, lethargy, loss of body condition, pyrexia, abdominal cramps and watery diarrhea. In severe cases, dehydration and cardiovascular collapse may occur leading to death in affected calves (6). Treatment options for cryptosporidiosis remain limited, with halofuginone lactate being the most widely used agent. Although it is not fully effective in curing the disease but its use has been associated to delay oocyst shedding and alleviating clinical signs. Furthermore, it also exhibits some degree of host cell toxicity which raises concerns about its safety to consume. Recently, pyrazolopyridine compound KDU731 has been reported to exhibit promising *in vitro* activity against *C. parvum* with minimal host cell toxicity and is being considered as a potential candidate for future therapeutic development (7).

Cryptosporidiosis is gradually attracting the attention of health professionals for being a zoonotic protozoan, mainly due to its connection with global water-borne epidemics. Due to its direct life cycle, parasite can spread via oral-fecal route, particularly through contaminated water and food (8). The oocysts of *Cryptosporidium* have been detected in run-off from agricultural areas, which consequently establish possible routes of transmission via contaminated recreational or drinking water. Such sewage contamination has been linked with outbreaks of cryptosporidiosis among humans and animals (9).

Oocysts of *Cryptosporidium* are shed in feces and spread through oral-fecal route via contaminated water or food. However, there are some genotypes/species which are distinctive to a particular host while the others persist as potent for zoonosis (6). Historically, the *C. parvum* was considered the sole species responsible for human cryptosporidiosis. However, the genetic characterization of parasite oocysts revealed the presence of two distinct species; *C*. *hominis* (genotype I; anthroponotic genotype), which is restricted to humans and *C*. *parvum* (genotype II; bovine/zoonotic genotype) which infects both humans and a wide range of animals (10). The most predominant zoonotic species among cattle is *C*. *parvum*, accountable for nearly 85% of the *Cryptosporidium* infections among pre-weaned calves (11).

Cryptosporidiosis can be detected in animals and humans using microscopic, immunological and molecular methods. Traditional microscopy is cheap but with low sensitivity and greatly reliant on shedding intensity of the oocysts and observer's skills. Molecular techniques like PCR are very sensitive and can be used to identify species and genotypes but these are expensive and laborious. Another alternative approach is copro-antigen detection using immunodiagnostic assays, particularly, direct immunofluorescence assay and enzyme-linked immunosorbent assay (ELISA), which enables rapid and high-throughput screening of fecal samples. The copro-antigen ELISA assays are widely used in epidemiological investigations of cryptosporidiosis and are considered reference diagnostic methods due to their balanced sensitivity, specificity, feasibility, and reproducibility (12, 13).

Nomadic pastoral communities of Pakistan depend largely on livestock production, and they have a close contact with animals (14). Livestock maintained under nomadic pastoral systems differ epidemiologically from animals kept in settled intensive/extensive dairy herds due to frequent herd movement, communal grazing, shared water sources, and limited access to routine veterinary services, which collectively increase their exposure to pathogens, including zoonotic pathogens (15). As a result, nomads are more likely to be infected with zoonotic pathogens including *Cryptosporidium* by cross-transmission across animals and human beings. Various factors including close contact with animals, food consuming behaviors, milk and meat contamination issues, tough environmental conditions and limited/no availability of drugs for treatment and traditional practices make nomadic people more exposed and thus prone to be infected with zoonotic pathogens (16). Moreover, lack of education and awareness regarding zoonotic pathogens, inadequate diagnostic facilities for zoonotic problems in basic health units are also important factors inducing the exposure of the nomadic pastorals to the zoonotic pathogens (14). Nomads are neglected communities and lack of focus of the health-related government institutions toward the nomad's leads to infections acquired from animals, animals' feces, and sewage material from their surroundings (17). The scientific data regarding the status of zoonotic infections among animals and human populations of marginalized nomadic communities is limited/scarce in most parts of the world including Pakistan. The wandering nomads and their animals are also a potential source of spreading zoonotic infection from one place to the other while migrating in search of food and other living requirements. Thus, nomadic communities are a permanent threat for direct or indirect introduction of various infections from one region to another. In this regard, it is direly needed to explore the status of zoonotic pathogens including *C*. *parvum*, especially in the livestock population along with associated risk factors to devise effective disease control and prevention strategies/policies for their containment in animals and thus to avoid their zoonotic transmission. Keeping in view, this study was conducted to determine the copro-antigen ELISA based prevalence of *C. parvum* and its associated risk factors in cattle population of nomadic communities located in Multan, an important agro-ecological zone of southern Punjab-Pakistan.

## 2 Materials and methods

### 2.1 The study area

**Figure 1:**
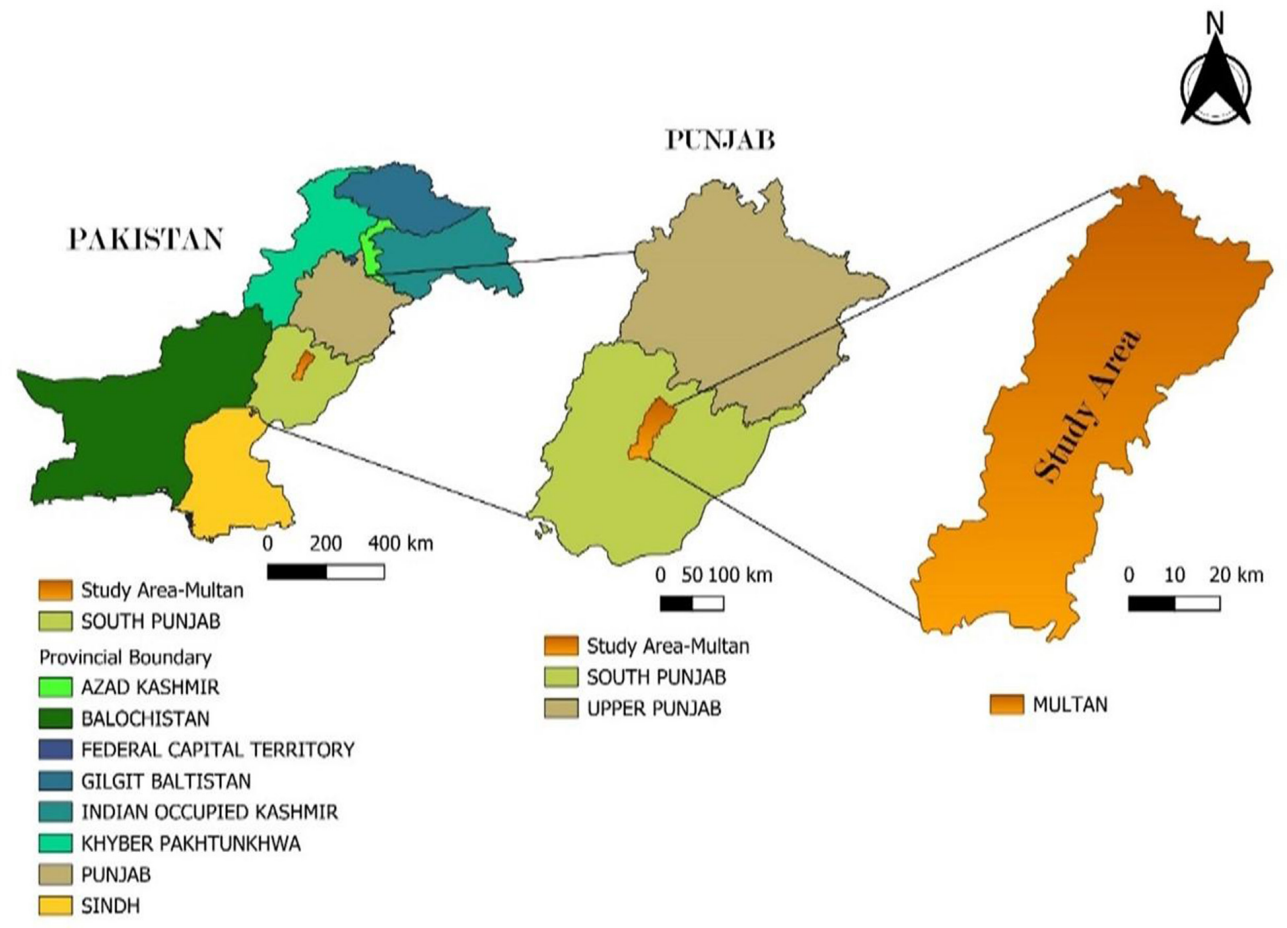
Map of study area showing agroecological zone of Multan-Pakistan (Developed using QGIS 3.24.2-Tisler, an open-source GIS software.

An analytical cross-sectional study was conducted in Multan region which is an important agroecological zone of southern Punjab, Pakistan (Figure 1). It is a highly populated region with high animal density. It has a total area of 3,721 Km^2^ with a population density of 1,302 individuals/km^2^. It is situated between 30.2 °N and 71.45 °E and is located at an altitude of 710 ft above sea level and is attached by Chenab River. Its land is very fertile and plain. However, during the monsoons season, the areas adjacent to the Chenab River are flooded. Large and small ruminants including buffaloes, cattle, goat and sheep are major livestock species being raised by the livestock farmers as a source of income and entrepreneurship. The climate of Multan is called desert climate with extremely hot summer (maximum 50 °C) and cold winter (minimum 1 °C) seasons. The average annual temperature and rainfall in Multan are 25.6 °C and 127 mm, respectively (18, 19).

### 2.2 Target population

The cattle raised by nomadic communities located in and around Multan region were targeted in this study to determine the coproprevalence of *C*. *parvum*. For the purpose, nomadic communities were mapped with the help of Livestock and Dairy Development (L&DD) Department, Govt. of the Punjab (Multan Division). The ruminants (bovines) are the main source of livelihood and wealth for nomadic communities. The nomads in the study area raised cattle, buffaloes, sheep, goats and camels as a source of their earning by the sale of animals and their products including milk and meat. The nomads have more frequent interaction with livestock species for different husbandry practices including feeding, drinking and removal/handling of their excreta; and spend 8–12 h/day with their animals. Additionally, nomads also share the same covered places/areas/sheds with their animals to live in and perform various other daily life activities. Moreover, these people make dung cakes from excreta of large ruminants to use them as a source of fuel to cook food and female nomads make these dung cakes bare-handed. Such a close and frequent interaction with animals and their excreta make nomads more prone to be infected with enteric zoonotic infections. The nomads are socio-economically deprived communities with poor sanitary and hygienic conditions. Being economically low resource class of society, nomads are unable to offer appropriate feed to their animals as per recommended feeding plan. Mostly animals fulfill their feeding requirements from grazing which are usually not safe for being contaminated with parasitic eggs/cysts. The poor husbandry and feeding management may negatively affect the immune functions of animals making them more susceptible to various infectious diseases including cryptosporidiosis. Accordingly, cattle population raised under nomadic set ups was targeted to explore the prevalence and associated risk factors of cryptosporidiosis, based upon which public health risk by its cross-transmission from cattle to human population may be assessed.

### 2.3 Collection of fecal samples

**Figure 2:**
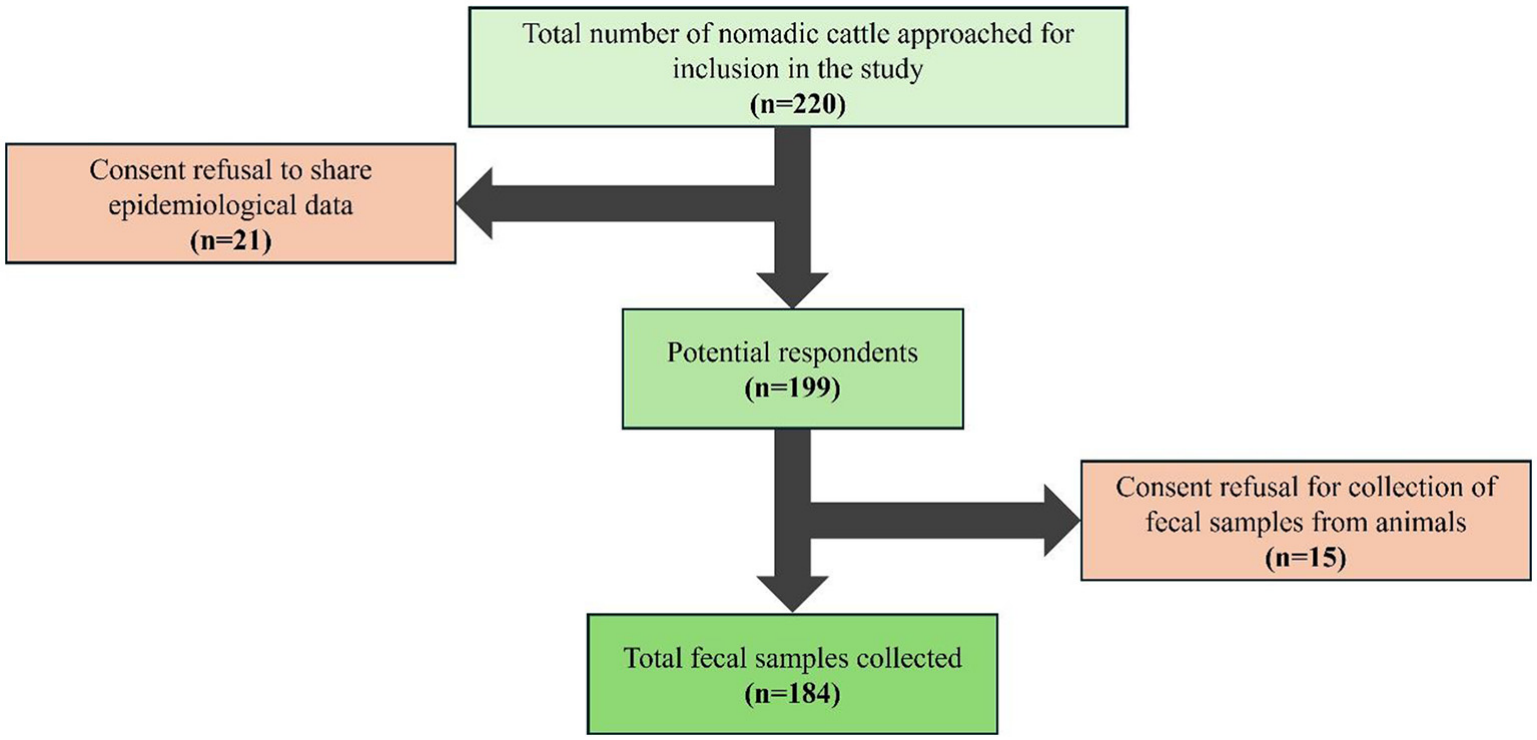
Scheme for inclusion of cattle in the study for sample collection.

For sample collection, 93 nomadic families/tribes who raised cattle were approached with the help of L&DD Department. The targeted communities were informed about the importance of zoonotic diseases along with possible impacts of these diseases on human health to enhance and ensure their interest and active participation in this study. Initially, a total of 220 cattle were selected through convenience sampling for inclusion in this study. However, due to lack of owners' consent, fecal samples were ultimately collected and analyzed from 184 animals (Figure 2). All the samples were collected directly from rectum in pre-labeled sterilized containers and were transferred to One Health Research Lab., Parasitology Section, Pathobiology Department, Bahauddin Zakariya University (BZU), Multan-Pakistan under cold chain and stored at −40 °C till further use.

### 2.4 Collection of descriptive epidemiological data regarding risk determinants

The descriptive epidemiological data regarding the possible risk determinants regarding gender, age, herd size, breed, clinico-physical appearance (assessed visually based on body condition score and symptoms), location, educational status of farmers, known history of GIT problem, source of drinking water, feeding pattern, physiological status of females, deworming history, hygienic condition (based on cleanliness of housing floors, frequency of manure removal, and visible fecal contamination), known contact with other wild-life species, presence of other *Cryptosporidium* susceptible animals and routine vaccination status of animals was recorded on pre-designed questionnaire (Supplementary material) to rule out the association of these factors with coproprevalence of cryptosporidiosis in target population.

### 2.5 Ethical approvals

Prior approvals regarding ethical concerns related to this study were obtained from the Board of Studies of Department of Pathobiology followed by Advanced Studies and Research Board of BZU, Multan (Edstt. No. Acad/MPhil/FVS/14-16/1178) before the initiation of this study. Prior verbal informed consent was also obtained from nomadic animal owners for using data generated from analyses of fecal samples of their animals for academic research and publication purposes in an anonymous manner.

### 2.6 Immunodiagnostic analysis of fecal samples using commercially available species-specific Copro-antigen ELISA kit

The copro-antigen of *C. parvum* was detected in fecal samples of target population using commercially available ELISA based species-specific *C. parvum* Antigen Test kits (Ref #: P00605-1; Lot No. 8038; Sensitivity = 98%; Specificity = 94%, IDEXX, USA). To ensure the reliability of results, all the samples were analyzed in duplicate. The test protocol was performed in accordance with the instructions of the manufacturer. The optical density values of samples and controls were measured at 450 nm using an ELISA Reader (ELx800, BioTek, USA) and data was interpreted using xChek^®^ 3.3 data analysis software (IDEXX, USA). Positive and negative controls supplied with the kit were included in each assay run and results were interpreted in accordance with the manufacturer's quality control recommendations.

### 2.7 Statistical analysis

The immunoassay-based and descriptive epidemiological data collected through the field survey were analyzed using bivariate and multivariable analysis in Minitab (Version 19.0) and R (Version 4.4.3). Chi-square tests, Fisher's exact tests, 95% confidence intervals (CIs), and odds ratios (ORs) were used to assess associations between different risk factor variables. All variables found to be significant in the bivariate analysis were included in a multivariable logistic regression model. A *P*-value of < 0.05 was considered statistically significant. The multicollinearity of variables in the multivariable logistic regression model was analyzed through generalized variance inflation factor (GVIF) values.

## 3 Results

### 3.1 Overall coproprevalence of cryptosporidiosis in cattle raised by nomadic communities

Results showed that out of 184 fecal samples, 25 were found positive for *C*. *parvum* by copro-antigen ELISA with an overall prevalence rate of 13.59% (95% CI: 9.03–19.21).

### 3.2 Association of cryptosporidiosis with host (cattle) related risk factors

The results regarding the association of cryptosporidiosis with host related risk factors in target population are shown in Table 1. In gender-wise analysis of data, prevalence rate of *C*. *parvum* antigen in male cattle was higher (17.65%) as compared to females (12.67%) however the difference was statistically non-significant (*p* = 0.444). The data collected from the field survey was categorized into four age groups viz. ≤ 1 year, >1 but ≤ 2 year, >2 but ≤ 3 years, >3 years. Highest prevalence rate was recorded in cattle aged ≤ 1 year (21.69%) whereas the cattle aged >2 but ≤ 3 years showed the lowest prevalence rate (5.26%). The difference in age-wise prevalence was statistically significant (*p* = 0.028). Cattle of four different breeds viz. Dhani, Desi Cross, Red Sindhi and Cholistani raised by the targeted nomadic communities were included in this study. The breed-wise analysis showed a non-significant association between cattle breeds and coproprevalence of cryptosporidiosis (*p* = 0.250). With respect to clinico-physical appearance, the data of study participants was categorized into three different groups including i) well-nourished/apparently healthy ii) malnourished/apparently weak iii) mild-moderate diarrheal animals to determine the correlation between clinico-physiological condition and prevalence of cryptosporidiosis in cattle. The cattle with mild-moderate diarrhea showed the highest (24.64%) prevalence followed by those of malnourished/apparently weaker (7.27%) and well-nourished/apparently healthy groups (6.67%) with statistically significant difference (*p* = 0.004). Similarly, the cattle with previous GIT problem(s) history also showed significantly (*p* = 0.016; OR = 4.92) higher prevalence rate (17.42%) as compared to those without any previous GIT problem(s) history (3.85%). With respect to physiological status of female animals, lactating cattle showed the highest prevalence (20%) followed by pregnant (16%) and non-pregnant cattle (2%) and the association was statistically significant (*p* = 0.018).

**Table 1:** Association of cryptosporidiosis with host (cattle) related risk factors in nomadic communities of Multan region of Pakistan.

Variable	% Prevalence (*n*/T) (95% CI)	% True prevalence (95% CI)	Chi-square test		Fischer's exact test (*P*-value)	Odd's ratio	Confidence interval (95%)	
			χ^2^	*P*-value			Lower limit	Upper limit
Gender								
Male	17.65 (6/34) (7.97–33.44)	12.66 (2.14–29.83)	0.586	0.444	0.42	1.50	0.50	3.95
Female	12.67 (19/150) (8.09–18.82)	7.25(2.27–13.94)				Ref		
Age (years)								
≤ 1	21.69 (18/83) (13.44–31.74)	17.05 (8.08–27.98)	9.083	0.028	0.033	Ref		
>1 but ≤ 2	5.40 (2/37) (0.96–18.10)	0.00^*^ (0.00–13.16)				0.21	0.2	0.47
>2 but ≤ 3	5.26 (2/38) (0.93–17.62)	0.00^*^(0.00–12.63)				0.02	0.02	0.08
>3	11.54 (3/26) (3.22–30.04)	6.02^*^ (0.00–26.13)				0.95	0.93	1.85
Breed								
Dhani	12.20 (5/41) (4.92–25.41)	6.73^*^ (0.00–21.10)	4.104	0.250	0.228	Ref		
Desi cross	19.15 (9/47) (10.07–32.67)	14.29 (4.42–28.99)				1.7	0.46	7.08
Red Sindhi	17.02 (8/47) (7.83–30.48)	11.98 (1.98–26.61)				1.47	0.38	6.27
Cholistani	6.12 (3/49) (1.68– 16.72)	0.13^*^(0.00–11.65)				0.47	0.07	2.62
Clinico-physical appearance								
Apparently healthy/well-nourished	6.67 (4/60) (2.31–16.14)	0.72^*^ (0.00–11.02)	11.131	0.004	0.005	Ref		
Apparently weak/malnourished	7.27 (4/55)	1.38 (0.00–11.98)				1.1	0.19	6.22
Diarrhea/mild–moderate	24.64 (17/69) (15.53–36.06)	20.26 (10.36–32.67)				4.53	1.36	19.7
History of GIT problem								
Yes	17.42 (23/132) (11.47–24.83)	12.42 (5.94–20.47)	5.858	0.016	0.0158	4.92	1.37	34.10
No	3.85 (2/52) (0.68–12.84)	0.00^*^ (0.00–7.44)				Ref.		
Physiological Status of female cattle								
Pregnant	16.00 (8/50) (7.29–28.63)	10.87 (1.40–24.60)	8.076	0.018	0.009	Ref.		
Non–pregnant	2.00 (1/50) (0.10–10.31)	0.00 (0.00–4.68)				0.11	0	0.87
Lactating	20.00 (10/50) (10.29–33.70)	15.22 (4.66–30.11)				1.31	0.42	4.24

^*^Negative true prevalence estimates were truncated to 0%. *P*-value < 0.05 is considered significant.

### 3.3 Association of cryptosporidiosis with animal husbandry related risk factors

Results revealed a significant association between herd size and prevalence of cryptosporidiosis (*p* = 0.030). The highest prevalence rate (22.58%) was recorded in cattle raised in a herd size with >10 heads whereas the lowest prevalence (6.67%) was recorded in herds with 1–5 heads. Drinking water source was also found to have significant association with coproprevalence of cryptosporidiosis in cattle (*p* = 0.028). The animals drinking water from stagnant water ponds showed the highest prevalence (20.45%) whereas the lowest prevalence was recorded in cattle drinking fresh tube-well/tap water (4.76%). Mode of feeding showed no association with cryptosporidiosis in cattle (*p* = 0.128). Regarding correlation of previous deworming history, it was revealed that cattle with previous deworming history had lower prevalence rate (10.62%) as compared to those without any deworming history (33.33%) and the difference was significant (*p* = 0.002). Similarly, a significant association was recorded between history of routine vaccination against endemic infectious diseases and prevalence of cryptosporidiosis (*p* = 0.017). Data analysis revealed a non-significant association between prevalence of cryptosporidiosis and contact of target population with wild animals (*p* = 0.164). In the target population, the association between hygienic conditions of housing facilities of animals and coproprevalence of *C. parvum* was found non-significant (*p* = 0.167); however as per our assumption, the animals housed under good hygienic conditions showed lower prevalence (5.26%) as compared to other groups with moderate (6.25%) and poor (16.54%) hygienic conditions. Results revealed significantly lower prevalence rates of cryptosporidiosis in cattle raised alone (3.70%) as compared to those raised with other *C. parvum* susceptible animals (17.69%; *p* = 0.012) (Table 2).

**Table 2:** Association of cryptosporidiosis with animal husbandry related risk factors in nomadic communities of Multan region of Pakistan.

Variable	% Prevalence (*n*/T) (95% CI)	% True prevalence (95% CI)	Chi-square test		Fischer's exact test (*P*-value)	Odd's ratio	Confidence interval (95%)	
			χ^2^	*P*-value			Lower limit	Upper limit
Herd Size								
1 to 5 heads	6.67 (4/60) (2.31– 16.14)	0.72 (0.00–11.02)	6.997	0.030	0.042	Ref		
6 to 10 heads	11.29 (7/62) (5.11– 21.39)	5.75 (0.00–16.73)				1.77	0.42	8.74
>10 heads	22.58 (14/62) (13.18–34.47)	18.02 (7.80–30.95)				4.04	1.17	17.98
Source of drinking water								
Tube-well/tap water	4.76 (2/42) (0.85– 15.92)	0.00 (0.00–10.79)	7.182	0.028	0.034	Ref		
Stagnant pond water	20.45 (18/88) (12.67– 29.93)	15.71 (7.25–26.01)				5.09	1.12	47.54
Canal water	9.26 (5/54) (3.71– 19.89)	3.54^*^ (0.00–15.10)				2.03	0.31	22.35
Feeding pattern								
Grazing	12.025 (19/158) (7.66– 17.87)	6.55 (1.80–12.90)	2.32	0.128	0.131	0.45	0.17	1.39
Grazing + stall feeding	23.08 (6/26) (10.55– 42.08)	18.56 (4.95–39.21)				Ref.		
Deworming history								
Yes	10.62 (17/160) (6.40–16.38)	5.03 (0.44–11.28)	9.166	0.002	0.006	0.24	0.09	0.67
No	33.33 (8/24) (16.90– 54.32)	29.71 (11.85–52.52)				Ref.		
History of contact with wildlife								
Yes	16.00 (20/125) (10.10– 23.41)	10.87 (4.45–18.93)	1.933	0.164	0.248	2.01	0.76	6.43
No	8.47 (5/59) (3.39– 18.19)	2.69^*^ (0.00–13.25)				Ref.		
Hygienic conditions of animals								
Poor	16.54 (22/133) (10.93– 23.89)	11.46 (5.35–19.44)	3.577	0.167	0.21	Ref.		
Moderate	6.25 (2/32) (1.12– 19.59)	0.27^*^ (0.00–14.77)				0.34	0.04	1.51
Good	5.26 (1/19) (0.26– 25.17)	0.00^*^ (0.00–20.83)				0.28	0.01	1.98
Contact with susceptible animals								
Yes	17.69 (23/130) (11.65– 25.21)	12.71 (6.14–20.88)	6.359	0.012	0.010	5.21	1.45	36.10
No	3.70 (2/54) (0.66– 12.37)	0.00 (0.00–6.92)				Ref.		
History of routine vaccination								
Yes	11.25 (18/160) (6.96– 17.02)	5.71 (1.04–11.97)	5.706	0.017	0.026	0.31	0.11	0.90
No	29.17 (7/24) (13.31– 50.00)	25.18 (7.94–47.83)				Ref.		

^*^Negative true prevalence estimates were truncated to 0%. *P*-value < 0.05 is considered significant.

### 3.4 Association of cryptosporidiosis with location and educational status of farmers

For this study, cattle were sampled from nomadic communities located in all four directions including north, south, east and west of Multan region. Although the prevalence was highest in cattle located in southern part (16%) and lowest in those of western part (10.20%) of Multan region however the statistical difference was non-significant (*p* = 0.822). Majority of nomads of targeted tribes were illiterate. However, a very small fraction had primary-middle qualifications as many NGOs are working on the well-being of nomads through field school education for poverty alleviation. The data collected from field survey was divided into three categories including illiterate, primary and above primary. The analysis revealed that there is no significant association of educational status of nomadic farmers with prevalence of cryptosporidiosis in cattle raised by them (*p* = 0.256) (Table 3).

**Table 3:** Association of cryptosporidiosis with miscellaneous Risk Factors in nomadic communities of Multan region of Pakistan.

Variable	% Prevalence (*n*/T) (95% CI)	% True prevalence (95% CI)	Chi-square test		Fischer's exact test (*P*-value)	Odd's ratio	Confidence interval (95%)	
			χ^2^	*P*-value			Lower limit	Upper limit
Location of animals								
North communities	12.5 (5/40) (5.06– 25.79)	7.06 (0.00–21.51)	0.914	0.822	0.821	Ref		
South communities	16.00 (8/50) (7.29– 28.63)	10.87 (1.40–24.60)				1.33	0.35	5.65
East communities	15.56 (7/45) (7.05– 28.58)	10.39 (1.14–24.54)				1.29	0.32	5.64
West communities	10.20 (5/49) (4.11– 21.94)	4.57^*^ (0.00–17.32)				0.8	0.17	3.77
Education status of nomadic farmers								
Illiterate	18.18 (12/66) (10.09– 29.28)	13.24 (4.45–25.30)	2.727	0.256	0.258	Ref		
Primary	8.57 (6/70) (3.79– 17.45)	2.80^*^ (0.00–12.44)				0.42	0.12	1.32
Above primary	14.58 (7/48) (6.61– 27.69)	9.33 (0.66–23.58)				0.77	0.23	2.35

^*^Negative true prevalence estimates were truncated to 0%. *P*-value < 0.05 is considered significant.

### 3.5 Multivariable analysis

Multivariable logistic regression was conducted to identify independent risk factors associated with cryptosporidiosis in cattle. The variables found to be statistically significant (*p* < 0.05) in the bivariate analysis were included in the model to maintain parsimony given the relatively high number of variables being studied. The variable “physiological status of females”, which was found to be significantly associated with cryptosporidiosis in bivariate analysis, was excluded from the multivariable model due to a high proportion of missing values, as it did not apply to male animals. Including this variable would have reduced the overall sample size and introduced sex-related bias. Therefore, to preserve model validity and interpretability, this variable was removed.

The final model included age, herd size, contact with susceptible animals, routine vaccination, water source, deworming history, health status, and history of gastrointestinal problems. All variables had generalized variance inflation factor (GVIF) values below the acceptable threshold [GVIF^(1/2Df)^ < 2], indicating no evidence of multicollinearity in the multivariable logistic regression model. The model demonstrated an excellent fit (McFadden's pseudo-R^2^ = 0.312; AIC = 128.66). Results revealed that age ≤ 1 year (OR = 7.79; 95% CI: 1.40–75.91; *p* = 0.04) and contact with susceptible animals (OR = 7.74; 95% CI: 1.72–61.97; *p* = 0.02) was significantly associated with higher odds of *C. parvum* copro-antigen positivity. Smaller herd size (1–5 heads; OR = 0.21; 95% CI: 0.04–0.82; *p* = 0.04) and routine vaccination (OR = 0.17; 95% CI: 0.04–0.67; *p* = 0.01) showed a significant protective effect (Table 4).

**Table 4:** Multivariate analysis of risk factors associated with cryptosporidiosis based on logistic regression on the significant variables selected by bivariate analysis.

Variable	Variable level	Regression coefficient	Standard error	adjusted Odds ratio	95% CI OR		*Z*-value	*p*-value
					Lower	Upper		
**Age (**years**)**	≤ 1	2.05	0.99	7.79^a^	1.40	75.91	2.07	0.04
	>1 but ≤ 2	Ref.						
	>2 but ≤ 3	−0.61	1.22	0.54	0.04	6.56	−0.50	0.62
	>3	1.64	1.17	5.15	0.56	63.76	1.41	0.16
**Clinico-physical appearance**	Apparently healthy/well-nourished	Ref.						
	Apparently weak/malnourished	−0.53	0.94	0.59	0.09	3.89	−0.56	0.58
	Diarrhea/mild-moderate	0.16	0.94	1.17	0.19	8.24	0.17	0.87
**History of GIT problem**	Yes	1.64	1.15	5.16	0.65	63.61	1.42	0.16
	No	Ref.						
**Herd size**	1 to 5 heads	−1.57	0.75	0.21^b^	0.04	0.82	−2.09	0.04
	6 to 10 heads	−0.68	0.62	0.51	0.14	1.68	−1.10	0.27
	>10 heads	Ref.						
**Source of drinking water**	Tube-well/tap water	0.87	1.23	2.38	0.19	25.87	0.70	0.48
	Stagnant pond water	1.11	0.66	3.04	0.90	12.15	1.70	0.09
	Canal water	Ref.						
**Deworming history**	Yes	−0.67	0.71	0.51	0.12	2.07	−0.94	0.35
	No	Ref.						
**Contact with susceptible animals**	Yes	2.05	0.88	7.74^c^	1.72	61.97	2.33	0.02
	No	Ref.						
**History of routine vaccination**	Yes	−1.79	0.71	0.17^d^	0.04	0.67	−2.51	0.01
	No	Ref.						

McFadden's pseudo-R^2^ = 0.318; AIC = 127.8. ^a^The odds of testing positive for cryptosporidiosis were 7.79 (95% CI = 1.4–75.91) times higher in cattle with age ≤ 1 year as compared to cattle with age >1 but ≤ 2. ^b^The herd size of 1 to 5 cattles exhibited protective odds (0.21; 95% CI = 0.04–0.82) of cryptosporidiosis. ^c^Cattles having contact with susceptible animals had increased odds of cryptosporidiosis by a factor of 7.74 (95% CI = 1.72–61.97). ^d^A history of routine vaccination decreased the odds of testing positive with cryptosporidiosis by 0.17 (95% CI = 0.04–0.67) times.

## 4 Discussion

*Cryptosporidium* is one of the most important causes of parasitic gastrointestinal disorder in various animal species. It results in significant economic losses in dairy sector (5). Additionally, it has also zoonotic implications and cross-transmission between animals and human beings is well-documented. Previous studies have demonstrated the zoonotic transmission of cryptosporidiosis between cattle and farm workers at different dairy farms (20). The only way to control the disease is to devise and implement effective control strategies based upon epidemiological data regarding prevalence patterns and risk factors of cryptosporidiosis in different parts of the world. These patterns vary widely due to regional differences in socio-economic status of farmers, geo-climatic conditions and animal husbandry practices (21). Therefore, this study was conducted to determine the coproprevalence and associated risk factors of cryptosporidiosis in cattle population of socio-economically deprived nomadic communities of Multan-Pakistan. These nomads bring their animals to live animal markets for sale and *C. parvum-*infected animals may facilitate the spread of infection to other animals and humans, thereby representing a significant veterinary and public health concern.

Findings of this study revealed that overall coproprevalence of *C*. *parvum* in cattle population of study area was 13.59%. A wide variation has been reported in the prevalence of *C*. *parvum* infection between different regions of the world. Contrary to our findings, higher prevalence rates of cryptosporidiosis had been reported in different parts of the world including 63.33% in Mardan, Khyber Pakhtunkhwa-Pakistan (22), 26.15% in Punjab, India (23), 67% at cattle herd level in Buenos Aires Province, Argentina (24), 57% in United Kingdom (25), 24.3% in Shandong-China (26) and 35.5% in East Coast of the US (27). On the other hand, lower prevalence rates had also been reported in various parts of the world viz. 1.9% in Lashkargah, Afghanistan (28), 9.2% in El-Behira, Egypt (29), 10.2% in England and Wales (30) and 2.6% in Eastern United States (31). Like our findings, some previous studies also reported comparable prevalence rates of in cattle population of Galicia, Spain (14.2%) and Ethiopia (15.8%) (32, 33). The variation in prevalence of *C. parvum* in different parts of world might be attributed to differences in the diagnostic methods used and study design. Socio-economic, geographical conditions and husbandry practices may also contribute to this variation. The higher prevalence rates particularly in developing and underdeveloped countries might also be correlated with poor sanitary/hygienic conditions and inappropriate healthcare facilities which aggravate the horizontal transmission of GIT pathogens including *C*. *parvum*.

Results showed a non-significant difference in gender-wise prevalence of *C*. *parvum* in target population and our findings are in line with previous studies conducted in Iran and south India (34, 35). In contrast, varied findings had also been reported in some previous studies (36). The differences in above-stated studies might be attributed to various factors including hormonal differences in both genders and husbandry practices for rearing both gender; anyhow, further studies are needed to explore this association. In nomadic production system, animals graze freely on communal pastures, resulting in similar levels of exposure to contaminated pastures for both sexes. It might be speculated as a reason behind non-significant difference in gender-wise prevalence of cryptosporidiosis in our study. Both bivariate and multivariable analysis revealed that age was significantly associated with coproprevalence of *C*. *parvum* in nomadic cattle population. Previously, similar findings had been reported in different parts of the world (24, 34, 35). The higher prevalence in young animals might be attributed to a less developed immune system and inability to resist against invading pathogens like *C. parvum*. Moreover, calves are usually infected at or soon after birth, because oocysts of parasite are shed in a fully sporulated and immediately infective form, facilitating rapid transmission in neonates.

Results revealed a non-significant association between coproprevalence of cryptosporidiosis and different cattle breeds. Previously, Venu *et al*. (35) also reported similar findings in a study conducted in south Indian dairy calves. In contrast, Gong *et al*. (37) reported a significant association of cattle breeds with the prevalence of *Cryptosporidium spp*. In this study, nomadic farmers had mixed farming of different breeds which were housed/kept together and grazed on same pastures. Co-farming of different breeds might be speculated for transmission of infection across different breeds and might be correlated with non-significant difference in breed-wise prevalence of cryptosporidiosis. Clinico-physical appearance of animals and previous history of GIT problems showed significant associations with cryptosporidiosis. In this study, the highest copro-antigen positivity (24.64%) of *C*. *parvum* was recorded in diarrheal cattle. Garro *et al*. (24) also reported higher coproprevalence of *Cryptosporidium* in diarrheal calves as compared to healthy ones. The altered mucosal immune responses in GIT disturbances including diarrhea might be correlated with high prevalence of *C*. *parvum* in the calves with mild to moderate diarrhea (4).

Among female cattle, the physiological status had significant association with cryptosporidiosis. The pregnant and lactating cattle had significantly higher prevalence rates (16%−20%) as compared to non-pregnant ones. Conversely, a previous study by Abdullah et al. (38) reported a non-significant association between pregnancy status and *Cryptosporidiosis* in cattle in Malaysia. It suggested that the influence of physiological status may vary and depend on other variables such as farming system, geographical setting, and management practices of the targtet population. Both bivariate and multivariable analysis revealed a significant association between herd size and coproprevalence of cryptosporidiosis in target population. To our information, no such direct correlation had been reported previously, however Garro et al. (24) reported prevalence ranging from 0 to 60% within-herd having different stocking density. Various factors such as close contact between animals due to high stocking density and unhygienic/dirty flooring in bigger herd size had been reported as high-risk factors for transmission of cryptosporidiosis (39). The analysis revealed a significant association between drinking water source and prevalence of cryptosporidiosis whereas association of feeding pattern was non-significant. The cattle drinking water from communal stagnant water ponds showed significantly higher prevalence rates as compared to those drinking water from other sources. In nomadic settings, the animals move freely and often have access to communal ponds which increases the risk of consuming water contaminated with parasitic cysts and/or eggs. These infected cattle in turn may contaminate other water bodies with cysts/eggs of parasites by defecation in those water bodies. Ultimately, such water bodies serve as a potential source of transmission of infection to humans and other healthy animals (8). On the other hand, a non-significant association with feeding pattern might be attributed to the fact that all the cattle population under investigation were grazing on open pastures with chances of cross-infection (through oral-fecal route) from each other while grazing on same pastures.

The cattle with history of deworming and routine vaccination against endemic diseases had lower prevalence rates and the difference was significant by bivariate analysis. However, multivariable analysis revealed only a significant association between routine vaccination and cryptosporidiosis. Deworming history might be correlated with cryptosporidiosis due to enhanced immunogenic efficiency of cattle to resist against invading pathogens including *C. parvum* by the elimination of GIT helminths by deworming and thus improved mucosal immunity (40). Regarding association of routine vaccination history, no data on such association exist in the available literature. However, it might be hypothesized that routine vaccination immunized the cattle against prevailing endemic infections, enabling them to perform better immunogenically to resist against other invading pathogens including *C*. *parvum* (41).

A non-significant association between prevalence of cryptosporidiosis and contact of target population with wild animals was observed in this study. However, Pumipuntu and Piratae (42) reported wild animals as potential reservoirs which may contribute in transmission of *Cryptosporidium* spp. to food, waters and other hosts. Nomads mostly live in open places at peripheries of urban areas and in these settings, there is no or minimal concept regarding maintaining proper separation between humans, domestic animals, and wildlife. Such close interactions and shared water sources can facilitate transmission of parasites across different host species.

The correlation between prevalence of cryptosporidiosis and housing facilities that were categorized as poor, moderate and good with reference to cleanliness was also non-significant. Mwaba *et al*. (39) also reported similar findings in livestock population of three districts of Zambia. In the nomadic livestock rearing system, animals remain untied for most of the day and are kept in confined housing facility for a few hours at night particularly in winter and rainy season. Therefore, the impact of cleanliness of housing facility is minimal in nomadic settings as compared to commercial farming system where animals are kept under confined conditions for most of the day. It might be speculated as a possible reason for the non-significant correlation of cleanliness of housing facility with prevalence of cryptosporidiosis in nomadic communities. The analysis regarding association between cryptosporidiosis and presence of other *C*. *parvum* susceptible animals (sheep/goat/buffalo) with the target cattle population was found to be significant by both bivariate and multivariable analysis. This might be attributed to the ability of parasite for cross-transmission across different species (10). The association between locations of targeted nomadic communities and cryptosporidiosis was non-significant. Literature revealed that geographical location might influence the pattern of prevalence of parasitic diseases due to difference in geoclimatic conditions and other factors (21). The non-significant association in this study might be attributed to relatively similar geo-climatic conditions and husbandry practices in the study area. The association between educational status of farmers and prevalence of cryptosporidiosis in cattle population was also non-significant. Previously no such association had been studied in animals for cryptosporidiosis however Khan et al. (43) reported a non-significant association between educational status of parents and prevalence of cryptosporidiosis in their children in a study in rural communities of Buner district of Pakistan.

## 5 Conclusions and recommendations

This study demonstrates that *C. parvum* is circulating among cattle raised by marginalized nomadic communities in an important agro-ecological zone of southern Punjab (Multan), Pakistan, with an overall copro-antigen–based prevalence of 13.59%. Multivariable analysis identified young age ( ≤ 1 year) and co-raising of other *Cryptosporidium*-susceptible animal species as significant risk factors for infection, whereas smaller herd size and routine vaccination against endemic diseases were associated with reduced odds of infection. These findings indicate that host susceptibility, animal mixing, and general herd health management play a central role in shaping the epidemiology of cryptosporidiosis in nomadic cattle systems. The detection of *C. parvum* in cattle maintained under extensive nomadic management highlights a potential public health concern, as close human–animal contact, shared water sources, and frequent animal movement may facilitate environmental contamination and zoonotic transmission. Given the mobility of nomadic populations, infected cattle may also contribute to the dissemination of the parasite across different geographic regions. Based on the identified risk factors, targeted control strategies should prioritize improving calf health, minimizing interspecies mixing where feasible, ensuring access to safe drinking water, and strengthening routine vaccination and basic veterinary service delivery within nomadic systems. Integrating nomadic communities into regional and national animal health and zoonotic disease surveillance programs is essential for effective control of cryptosporidiosis and other neglected zoonoses. Awareness-raising initiatives and mobile veterinary and public health services should be reinforced to reduce infection risk at the human–animal–environment interface.

## 6 Study limitations

This study has some limitations that should be considered when interpreting the findings. Firstly, the sampling was based on convenience due to the highly mobile nature of nomadic communities and the absence of formal animal registries, which limited the feasibility of random sampling. As a result, the findings may not be fully generalizable to all nomadic cattle populations within Punjab or other regions of Pakistan. In addition, sample size calculation was not performed as baseline prevalence estimates for cryptosporidiosis in target population were unavailable and lack of data frame/ animal registries restricted systematic enrollment. Consequently, the final sample size was determined by accessibility and owner consent. Secondly, the cross-sectional study design captures infection status at a single time point and therefore does not allow assessment of temporal or seasonal variations in *Cryptosporidium* transmission dynamics, which may influence prevalence estimates. Thirdly, detection of *C. parvum* was based on a copro-antigen ELISA. Although the diagnostic kit used has high reported sensitivity and specificity however misclassification bias cannot be completely excluded, particularly in cases of low antigen shedding or early infection. Additionally, molecular confirmation and genotyping were not performed due to limited laboratory resources, which restricted species-level confirmation and prevented evaluation of genetic diversity, transmission pathways, and zoonotic linkage between cattle and human infections. Finally, some explanatory variables relied on owner-reported information, such as previous gastrointestinal illness, deworming, and vaccination history, which may be subject to recall bias. Despite these limitations, the study provides important baseline epidemiological data on cryptosporidiosis in an understudied and high-risk nomadic livestock production system and identifies key management-related factors that can inform targeted disease control strategies.

## Data Availability

The raw data supporting the conclusions of this article will be made available by the authors, without undue reservation.
