# Supplementary material for: Status of cryptosporidiosis in cattle raised by marginalized nomadic communities of Southern Punjab, Pakistan: an observational study
Source: Front Vet Sci. 2026 May 27;13:1820598. doi: 10.3389/fvets.2026.1820598 (PMC13250939; doi:10.3389/fvets.2026.1820598)
Supplement: Supplementary file 1 [file Data_Sheet_1.pdf]

**Project:** Prevalence of *Cryptosporidium parvum* in the livestock population of nomadic communities of Multan, Southern Punjab, Pakistan

## PRE-DESIGNED QUESTIONNAIRE TO RECORD DESCRIPTIVE EPIDEMIOLOGICAL DATA

| | | | | |
|---|---|---|---|---|
| Serial/Sample No. | | Date of Sampling | | |
| Name of the nomadic farmer | | | | |
| Location/site of the farmer | | | | |
| Breed of the animal | | | | |
| Age of the animal | ≤1 year | >1 but ≤2 | >2 but ≤3 | >3 years |
| Gender/Sex of the animal | Male | | Female | |
| Herd size | 1 to 5 heads | 6 to 10 heads | >10 heads | |
| Clinico-physical appearance /Health status of animal | | | | |
| History of GIT problem | Yes | | No | |
| Source of drinking water | Tube-well/Tap Water | Stagnant Pond Water | Canal Water | Other, specify pl. |
| Feeding pattern | Grazing | | Grazing + Stall feeding | |
| Physiological status of female animals | Pregnant | Non-Pregnant | Lactating | |
| Deworming history | Yes | | No | |
| Hygienic condition of animals | Poor | Moderate | Good | |
| Known contact with other wild-life species | Yes | | No | |
| Presence of other *Cryptosporidium* susceptible animals | Yes | | No | |
| Routine vaccination History | Yes | | No | |
| Educational status of Nomadic Farmer | | | | |
| Any other factors found to be important with respect to specific geographical region and ritual/cultural values of study area | | | | |

Department of Pathobiology, Faculty of Veterinary Sciences
Bahauddin Zakariya University, Multan
